# The role of motor effort on the sensorimotor number system

**DOI:** 10.1007/s00426-024-02002-2

**Published:** 2024-07-09

**Authors:** Alessandro Benedetto, Eleonora Chelli, Irene Petrizzo, Roberto Arrighi, Giovanni Anobile

**Affiliations:** 1 Department of Neuroscience, Psychology, Pharmacology and Child Health, University of Florence, Florence, Italy; 2 Center for Mind/Brain Science, University of Trento, Rovereto, Italy; 3 School of Psychology, The University of Sydney, Sydney, Australia

## Abstract

The integration of numerical information with motor processes has emerged as a fascinating area of investigation in both animal and human cognition. The interest in a sensorimotor number system has recently generated neurophysiological and psychophysical evidence which combine to highlight the importance of motor functions in the encoding of numerical information. Nevertheless, several key questions remain, such as the influence of non-numerical motor parameters over numerical perception. Here we tested the role of physical effort, a parameter positively correlated with the number of actions, in modulating the link between hand-actions and visual numerosity perception. Effort was manipulated during sensorimotor adaptation as well as during a new actions-estimation paradigm. The results of Experiment 1 shows that physical effort in the absence of actions (passive effort) is not sufficient to activate the sensorimotor number system, indicating that self-produced actions are instead necessary. Further experiments demonstrated that effort is marginally integrated during motor adaptation (Experiment 2) but discarded when estimating the number of self-produced hand actions (Experiment 3). Overall, the results indicate that the sensorimotor number system is largely fed by the number of discrete actions rather than the amount of effort but also indicates that effort (under specific circumstances) might be integrated. These findings provide novel insights into the sensorimotor numerical integration, paving the way for future investigations, such as on its functional role.

## Introduction

Humans share with many animals a visual number sense, the non-verbal ability to estimate, roughly but quickly, the numerosity of objects in space and events in time (Dehaene, 2011). In the past few decades much research has been dedicated to this sensory ability as well as the underlying neurophysiological mechanisms. More recently, the concept of numerosity has been extended to the motor domain, leading to the idea of a sensorimotor number system encoding numerical information of both external sensory inputs as well as internally generated (self-produced) motor routines (Anobile et al., 2016, 2021, 2024).

The first evidence for motor number neurons comes from a seminal paper by Sawamura et al. (2002). The authors trained monkeys to make five movements (e.g., turn, push), repeating a movement then switching to another movement in a cyclical fashion. Neurons in the posterior parietal cortex showed selectivity to the number of self-generated actions, demonstrating the existence of neurons keeping track of the number of actions. However, as the animals were trained to perform a fix number (five) of actions that were not triggered by sensory information, this study did not provide information about the possible mechanisms of sensorimotor number transformation. Years later, Kirshhock and Nieder (2022) discovered, in the crow’s brain, sensorimotor number neurons whose activity was related to the translation of numerical visual inputs into action sequences. The animals were trained to peck for a specific number of times to match the number of visually presented digits or dots arrays (1–5). The authors discovered neurons in the telencephalon that were tuned to the impending number of self-generated actions during the phase between the disappearance of the target number and the onset of the motor reproduction (programming phase). The activity of these neurons predicted the behavioral performance (under- or over-estimation) and was independent of stimulus format (dots or digits). Each tuning function peaked at a given preferred number, with activity scaling down with numerical distance. Indeed, these cells in the crow’s brain could constitute the neural substrate which promotes the transformation of sensory inputs into a given quantity of numerical actions. In a subsequent study, Kirshhock and Nieder (2023), by leveraging on the same sensorimotor transformation task, demonstrated that crows’ performance on number production follows Weber Law, since the variability of their responses scaled proportionally with stimuli magnitude. Weber Law is a behavioral hallmark of the number sense, previously applied to the judgment of visual numerosity in animals (Ditz & Nieder, 2015; Kirschhock & Nieder, 2023; Nieder, 2016, 2020; Nieder & Miller, 2003) and humans (Anobile et al., 2014; Dehaene, 2011; Feigenson et al., 2004; Piazza, 2010; Ross, 2003).

There is also psychophysical evidence in humans supporting the existence of a sensorimotor number system. The first has been gathered by means of adaptation (Anobile et al., 2016). This technique leverages on a well-known physiological mechanism: when neurons are stimulated over a relatively long period of time (adaptation phase), their activity robustly decreases to alter the responsivity of the neural units tuned to the adapted feature (Barlow & Hill, 1963; Thompson & Burr, 2009). Therefore, when a new stimulus is subsequently presented around the adapted region, its perception will be distorted by the functional changes induced by the adaptor. For example, as noted by Addams back in 1834, after observing for a few seconds the downward movement of water in a waterfall, moving the gaze towards the surrounding rocks causes them to be perceived as moving upwards, even though they are physically still (Addams, 1834). It is to be noted that the adaptation phenomenon is important not only because it creates strong sensory illusions but also because it reveals important information about the organization of the sensory mechanisms underlying a given perceptual process. For example, the waterfall illusion is well accounted for in terms of the existence of neurons in the visual brain areas selectively tuned to up- and downward motion so that when one of these channels get adapted, the following stimulus is perceived as moving opposite to the adapting direction (Barlow & Hill, 1963). In other words, selective adaptation implies the existence of neurons tuned to that feature (Blakemore & Campbell, 1969; Clifford & Rhodes, 2005; Thompson & Burr, 2009). By leveraging on this idea, Anobile et al. (2016) devised a visuomotor number adaptation paradigm. During adaptation, participants had to tap (up/down) with their dominant hand in mid-air, below a screen, for a few seconds. After completing this sequence of actions, participants had to judge the numerosity of a briefly presented dot array or a temporal sequence of flashes displayed near the tapping (adapted) region. As predicted, due to adaptation, visual numerosity was underestimated after the production of “many” motor routines, while overestimated after “few” actions. The hand-tapping adaptation effect has been proved consistent and generalized: its effect on spatial visual numerosity (dot arrays) is independent of the spatial configuration of the visual items (Yang et al., 2024) and has been also found to affect numerosity perception of auditory sequences of tones in both sighted and congenitally blind adults (Togoli et al., 2020). Moreover, this effect has been proved to be genuinely generated by a modification of sensorimotor processes and not by post-perceptual processes such as response or decisional biases (Maldonado Moscoso et al., 2020).

Another piece of evidence for the existence of sensorimotor number mechanisms come from a psychophysical technique based on inter-individual differences (Anobile et al., 2024). This technique has been widely used in the past to reveal visual channels for basic sensory features such as motion (Morrone et al., 1999), spatial frequency (Reynaud & Hess, 2017; Simpson & McFadden, 2005), contrast sensitivity (Peterzell & Teller, 1996; Peterzell et al., 1995) and color (Peterzell & Teller, 2000; Peterzell et al., 2000). The hypothesis was the existence of a set of sensorimotor channels, each with a specific preference for a preferred number of actions/objects and organized as overlapping Gaussian distributions, like those described in crows (Kirschhock & Nieder, 2022). Such a system should be present and similarly organized in everyone. If such tuning channels exist, performance measures of stimuli detected by the same channel (neighboring numerosities) should correlate more between individuals than stimuli detected by different channels (numerically distant numerosities). To measure the sensorimotor number system, participants were presented with digits and required to rapidly press a key as many times as the target number, without counting [a sensorimotor number transformation task, like that used with crows by Kirschhock and Nieder (2022, 2023)]. Reproduction precision (Weber Fraction) was then correlated for various target numbers between participants. As predicted, the results showed high positive correlation for nearby target numbers encoded by neighboring channels and scaling down with numerical distance, implying tuning selectivity. A principal component analysis revealed two bell-shaped covariance channels, peaking at different numerical values. These results provided the first description of sensorimotor number channels in humans responsible for translating symbolic numbers into action sequences.

Overall, the psychophysical results, together with the discovery of motor and sensorimotor number cells and the behavioral parallelism between sensorimotor and visual numerosity performance in crows, suggest a unified sensorimotor number representation system underlying the judgment of the number of external stimuli and internally generated actions. However, the study of this new multisensory (action and perception) system has just started, and several questions are still open, some of them fundamental. One of these is related to the role of non-numerical motor parameters, such as physical effort. Performing a long sequence of actions involves more physical effort compared to performing relatively few actions. As this parameter is positively correlated with the number of actions, it could be usefully integrated and used by the system to provide, respectively, the sensory distortions induced by “many” (corresponding to high effort) and “few” (corresponding to low effort) actions in the motor adaptation technique exploited in the previous studies (Anobile et al., 2016; Maldonado Moscoso et al., 2020; Togoli et al., 2020; Yang et al., 2024). Going beyond this specific case of adaptation, whether the sensorimotor number system is number-specific, or it also exploits the effort of the action is a fundamental, and still unexplored, question.

To fill this gap, in the current study we focus on the role of this non-numerical motor parameter. More specifically, we assessed three main questions, each of which associated with a psychophysical experiment. First: is hand passive effort (in the absence of any actions) sufficient to affect visual (spatial) numerosity perception (Experiment 1)? Second: does the sensorimotor number adaptation effect on perceived visual numerosity (see above) depend on the effort associated with the hand actions performed during the adaptation phase (Experiment 2)? Third: is the effort associated with hand movements capable of shaping the perceived number of self-produced hand actions in the absence of any prior sensory instructions (Experiment 3)?

**Fig. 1:**
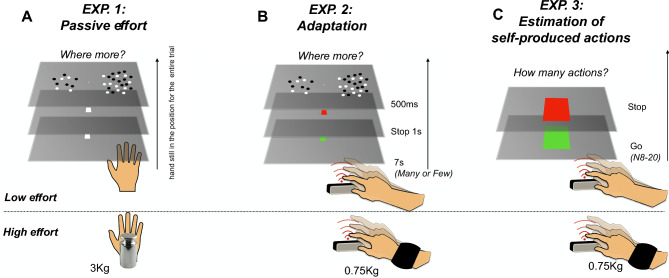
Schematic representation of the three experiments. **A** Experiments 1 (passive effort): on each trial, participants hold their dominant hand steady below a screen while judging which one of two dot arrays appear more numerous (2AFC task). In separate blocks, the discrimination task was performed holding (high effort) or not wearing (low effort) a 3 kg weight. **B** Experiment 2 (adaptation): On each trial, participants adapted with their dominant hand to a temporal sequence (7 s) of self-produced slow (few) or fast (many) mid-air tapping. In separate blocks, the adaptation phase was performed wearing (high effort) or not wearing (low effort) a 0.75 kg wristband. After the motor adaptation phase (indicated by the color change of the fixation point from green to red), a 1 s pause occurred, followed by the presentation of two dot arrays (500 ms). After the stimulus presentation, the participants were asked to verbally indicate the more numerous (2AFC discrimination task). **C** Experiments 3 (estimation of self-produced actions): on each trial, participants performed a sequence of few self-produced mid-air taps with their dominant hand below a screen. The taps were monitored by a motion-tracking device. When the number of taps reached the target number (8–20 in steps of 2, randomly selected trial by trial) the fixation point turned red, and the participants required to stop the motor routine and verbally report the number of self-produced actions

In Experiment 1 we manipulated the physical effort by asking participants to passively maintain their dominant hand suspended in mid-air, either while holding a 3 kg weight (high effort) or not (low effort). In Experiments 2 and 3 we manipulated the physical effort associated with the actions by applying a 0.75 kg weighted wristband to the wrist of the tapping hand (high effort) and matched the results with the condition in which the same action was performed with no additional weight (see Fig. 1). The hypotheses are straightforward. Experiment 1: if the sensorimotor system responds to effort together with or instead of action’s number, passive effort even in the absence of any actions was predicted to be sufficient to induce distortions of visual numerosity estimates. Experiment 2: if the sensorimotor system integrates the magnitude of physical effort in the encoding of self-produced actions, we expected different adaptation effects in the low and high effort conditions. As the number and effort of each action are positively correlated, effortful actions might be counted as “more numerous” compared to non-effortful actions. If this is the case, we predicted a modulation of the adaptation magnitude as a function of physical effort. In normal (low) physical effort conditions (i.e., without the application of additional weights), it has been previously shown that after an adaptation phase to few actions, physical numerosity is overestimated. In this case, the overestimation effect induced by an adaptation phase to a few but strenuous actions (possibly considered by the system as more numerous) should be weaker. Conversely, motor adaptation induced by the execution of numerous routines performed with additional weight on the hand should strengthen the numerosity underestimation previously reported in typical (no hand weight) conditions. Experiment 3: following the same rationale as above, if physical effort is integrated with the number of actions, we expected participants to overestimate the number of strenuous self-produced actions (performed with the hand weight) compared to the condition in which actions were non-strenuous.

## Materials and methods

### General procedure

Participants stood in a dark and quiet room facing a gamma-linearized monitor (LG Flatron L1730SF, 17’’, 60 Hz) positioned 44 cm below their head. The position of the dominant hand was continuously monitored by an infrared motion-tracking device (Leap Motion controller, San Francisco, CA, USA) running at 60 Hz, placed below the monitor. The study comprises 3 experiments: Experiment 1 studied the contribution of passive effort on visual numerosity perception when no sequential motor routines are carried out, Experiment 2 aimed at investigating the effect of effort on motor adaptation (sequential actions), Experiment 3 explored the influence of effort on the numerosity estimation of self-produced hand actions.

### Participants

Fifteen participants, with either normal or corrected-to-normal visual acuity, were recruited for Experiment 1 and 2. The group included 7 females and 8 males, with a mean age of 26.5 ± 3.98 years (mean ± standard deviation). Fourteen participants were right-handed, one left-handed. Sixteen participants took part in Experiment 3 (7 females, 9 males, mean age 27.4 ± 5.02, thirteen right-handed and three left-handed). Among these, 13 volunteers participated in all three experiments. All participants provided written informed consent, and the study received approval from the local ethics committee ("Commissione per l’Etica della Ricerca," University of Florence, 7 July 2020, n.111).

### Visual stimuli

Visual stimuli were generated using Psychophysics Toolbox for MATLAB (Brainard, 1997; Watson & Pelli, 1983). For Experiment 1 and 2 stimuli consisted of two arrays of dots, each of which subtending 0.3° in diameter. To balance the overall luminance of the set with the mid-gray background, 50% of dots were black, and 50% white (100% contrast). Each dot was displayed in random positions within a 20° diameter circle centered 11° to the left and right relative to a central fixation point (square, 0.3° in diameter). Dots were not overlapping, and center-to-center separated by at least 0.45°. To make the two arrays appear homogeneous in terms of area, two rules were applied to the generation of stimuli: (i) the area of each array (estimated as its convex hull) had to be greater than or equal to half the area of the circle within which the array was inscribed (i.e., $a\ge \frac{\pi {r}^{2}}{2}$, where $a$ is the convex hull of the array, and $r=6^\circ$ is the radius of the circle in which the stimulus is inscribed); (ii) the average radius of all dots in each array (relative to the center of the circular array itself) had to be less than 5° (where the maximum possible radius was 6°). Of the two arrays, one was referred to as the “reference” stimulus, always displaying 24 dots and presented in the spatial congruence with the dominant hand side. The second array was the “test” stimulus, presented on the opposite side and changing numerosity – trial-by-trial – between 12 and 36 dots (grain of 1), according to an adaptive QUEST procedure (Watson & Pelli, 1983).

### Experiment 1: passive effort

Experiment 1 consisted of a 2AFC numerosity discrimination task, with a within-subject block design comprising two physical effort levels (“low” and “high”). On each trial, a white central fixation point was presented for 500 ms. After this phase, two visual stimuli were simultaneously presented for 500 ms, one on the left and one on the right of the fixation point. After the visual stimuli disappeared, participants were asked to verbally report which array appeared to be more numerous (left or right). Participants were asked to hold their dominant hand steady in the reference position, below the screen, for the whole duration of the block. In the low effort condition, participants followed the procedure described above while in the high effort condition, the same was carried out with a 3 kg weight tied to their wrist. The two effort levels were tested on separated days and presented in a pseudo-randomized order. For each condition, participants performed two blocks of 35 trials (70 trials per condition, 140 trials in total). The proportion of “test more numerous” responses was plotted against the stimuli magnitude (in log scale) and fitted with a cumulative Gaussian error function. The 50% point of the fit provided an estimate of the point of subjective equality (PSE). A schematic representation of the paradigm is depicted in Fig. 1A.

### Experiment 2: motor adaptation and effort

As in Experiment 1, Experiment 2 consisted of a 2AFC numerosity discrimination task, with a 2 × 2 within-subject block design comprising two motor adaptations conditions − “few” and “many” actions − and two physical effort levels, “low” and “high”. After a preliminary phase in which participants familiarized themselves with the setup and the task, the experiment started with a motor adaptation phase. During this phase participants kept fixation on a green point while performing mid-air tapping movements for 7 s. The tapping movements were “up-down” movements of the dominant hand performed below the screen, with the hand concealed by the monitor in a position that (from the participant’s point of view) was spatially overlapping with the region where the reference stimulus would later appear. If the participant’s hand was not placed in the correct position, the trial was automatically aborted and repeated. The experiment comprised two levels of motor adaptation, tested in separated blocks, and presented in a pseudo-randomized order: adaptation to “few” actions, in which participants were instructed to tap continuously but slowly; adaptation to “many” actions, in which participants were asked to tap as fast as they could. After 7 s of tapping, the color of the fixation point turned from green to red, indicating the end of the adaptation phase when participants had to stop the in air tapping and hold their hand steady in the reference position. After a 1 s pause, the two visual stimuli were simultaneously presented for 500 ms, and participants asked to verbally report which array appeared to be more numerous (left or right). The response was recorded by the experimenter, blind to the stimuli. In addition to the two levels of motor adaptation, we also tested two levels of physical effort: in the low effort condition, participants followed the procedure described above; in the high effort condition, the same routine was carried out with a 750 g weight tied to the subject's wrist to make to the tapping routine more laborious. The two effort levels were tested in separated days and presented in a pseudo-randomized order. For each condition, participants performed two blocks of 35 trials (70 trials per condition, 280 trials in total). The proportion of “test more numerous” responses was plotted against the stimuli magnitude (in log scale) and fitted with a cumulative Gaussian error function. The 50% point of the fit provided an estimate of the point of subjective equality (PSE). A schematic representation of the paradigm is depicted in Fig. 1B.

### Experiment 3: estimation of self-produced actions

Experiment 3 consisted of a new “action-numerosity” estimation task, with a within-subject block design comprising two physical effort levels (“low” and “high”). Participants kept fixation on a green square while executing comfortably slow mid-air tapping movements below the screen (corresponding to “few actions” in the motor adaptation from Experiment 2). The online monitoring of tap count by the motion-tracking device determined the cessation of tapping signal, indicated by the change in color of the fixation point from green to red. The required number of taps varied randomly from trial to trial within the range 8–20 in steps of 2. Subsequently, participants were required to verbally estimate the number of taps they produced. To prevent explicit counting, participants engaged in subvocalizations throughout the tapping duration (repeating the syllable “ba”). Each tested condition comprised one block of 40 trials (80 trials in total, 6.5 ± 0.5 trials per numerosity per subject). A short training session preceded the execution of the experiment (2 repetitions for each numerosity to be tested). During this training, participants received trial-by-trial feedback on the physical number of produced taps. For each target number, we measured the average response (the accuracy across trials). The data collected in the training phase were not included in the main analyses. A schematic representation of the paradigm is depicted in Fig. 1C.

### Statistical analyses

Data were evaluated by means of Bayesian statistics (Bayesian t-test and repeated measure ANOVA), measuring Bayes Factors, the ratio of the likelihood of the alternative to the null hypothesis (i.e., BF10), and reporting them as base ten logarithms (hereafter LBF) (Jarosz & Wiley, 2014; Lavine & Schervish, 1999). For repeated measure ANOVA, we report both the model comparison analysis as well as the analysis of effects across all matched models. For model comparison, models were compared to the null model and—for all repeated measure factors—included subject and random slopes. For the analysis of the effect, we report the “LBF *inclusion*” probability, a measure of how much the data is likely to occur from a model including that specific factor or interaction. By convention, LBF > 0.5 is considered substantial evidence in favor of the alternative hypothesis and LBF < − 0.5 substantial evidence for the null hypothesis (Jarosz & Wiley, 2014). Absolute values greater than 1 are considered strong evidence, and greater than 2 definitive.

## Results

### Experiment 1: passive effort

**Fig. 2:**
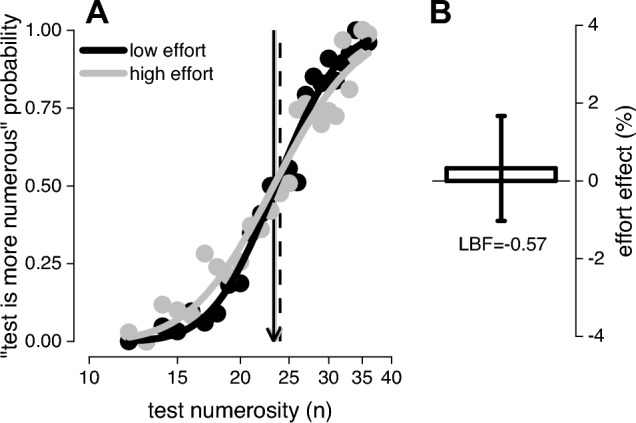
Passive effort on visual numerosity perception. **A** Psychometric curves (for aggregate data) measured while holding (high effort, light grey) or not (low effort, black) a 3 kg weight. **B** Between participants effect of passive effort on PSEs. Error bars reports ± 1 s.e.m

It has been previously demonstrated that after a prolonged period of hand taps (adaptation), the perceived numerosity of a dot array presented near the spatial region where the hand was tapping is biased by the number of actions performed during the adaptation phase: the perceived numerosity is underestimated after numerous actions and, conversely, overestimated after few actions (Anobile et al., 2016; Maldonado Moscoso et al., 2020; Togoli et al., 2020; Yang et al., 2024). However, since many actions typically requires the consumption of more energy, and thereby higher physical effort compared to few actions, in Experiment 1 we tested whether passive effort, the absence of actions, is sufficient to bias visual numerosity discrimination. To this aim, we had participants discriminate the relative numerosity of dot arrays while keeping one hand still in mid-air (under the screen) in spatial congruence with the reference stimulus. Effort was manipulated by applying (or not) a 3 kg weight on the steady hand while participants discriminated the numerosity of two patches of dots, one of which was presented spatially aligned with the hand and the other was presented in a diametrically opposite position on the left. If passive effort is sufficient to induce a distortion in visual perception, we expect the psychometric curves measured with (high effort) and without (low effort) the weight to spread apart. Figure 2 shows that this was not the case, with the two psychometric curves (for aggregate data) being almost completely superimposed. To quantify the effect, for each participant, we calculated an index as the difference between PSEs measured in the two effort conditions, normalized by the sum of the two. On average the effect was very small 0.3 ± 1.3% (Fig. 2B) and not different from zero (Bayesian 2-tailed t-test: LBF = − 0.57).

### Experiment 2: motor adaptation and effort

The results obtained from Experiment 1 indicate that passive effort, in the absence of any actions, is not sufficient to distort visual numerosity perception, suggesting a preferential link between numerosity and the action system. However, this null result does not rule out the possibility of an interaction between effort and action. In other words, when actions are in play, effort might still be coded and integrated with numerosity information. To this aim we investigated whether the motor adaptation effect depends on—or is affected by—the physical effort associated with the actions performed during the motor adaptation phase. We replicated the classical motor-adaptation paradigm on visual numerosity (Anobile et al., 2016) but also included a manipulation of the physical effort. The manipulation was achieved by having participants wearing or not wearing a 0.75 kg wristband on their tapping hand (corresponding to high effort and low effort condition, respectively), during the adaptation phase (see “Materials and methods” for details).

**Fig. 3:**
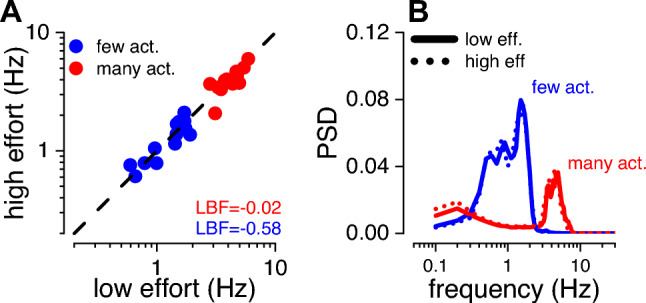
Motor parameter during the adaption phase. **A** Tapping rate (Hz) in the high (y-axis) versus low (x-axis) effort conditions, while participants were adapting to few (blue) or many (red) actions. Each dot represents a participant. **B** Power spectrum density (PSD) of the tapping profile (vertical motion) as a function of tapping frequency in the low (continuous lines) and high (dotted lines) conditions divided by when participants adapted to few (blue) or many (red) actions

As wearing or not wearing the wristband could in principle affect the action’s dynamic, such as the tapping rate and/or amplitude, we first compared the kinematic of the action across the two effort conditions. Figure 3A shows the tapping rate during the adaptation phase, separately for when participants adapted to “few” (blue symbols) or “many” (red symbols) actions. The tapping rates in the two conditions were clearly separated, confirming that participants performed more actions in the fast condition than the slow condition (tapping rate: 1.3 ± 0.11 and 4.08 ± 0.22 Hz, for few and many actions, respectively; 2-tailed paired t-test: LBF = 15.37). More importantly for the aim of the current study, most of the datapoints scattered along the equality line (dashed line), indicating similar tapping rates between the two effort conditions (high and low). In fact, the tapping frequency during the adaptation to few actions was 1.35 ± 0.11 Hz and 1.34 ± 0.12 Hz (mean ± 1 s.e.m.), for the low and high effort conditions respectively (2-tailed paired t-test: LBF = − 0.58). In trials following adaptation to many actions, tap frequency was 4.20 ± 0.22 Hz and 3.96 ± 0.22 Hz (mean ± 1 s.e.m.), in the low and high effort conditions respectively (2-tailed paired t-test: LBF = − 0.023). To control for differences in the action kinematics, we also analyzed the power spectra of the tapping profiles. Figure 3B shows its average power spectrum density (PSD) as a function of tapping frequency. Adapting to few actions (blue curves) generally resulted in higher power compared to many actions indicating that the faster the tapping, the smaller the vertical movements (up-down) were. Importantly, this was true for both low and high effort conditions. As evident in Fig. 3B, the spectra associated with the two effort conditions (continuous and dotted lines, for low and high effort condition, respectively) were almost perfectly superimposed, indicating that the kinematics of the movement was well preserved across different effort conditions.

**Fig. 4:**
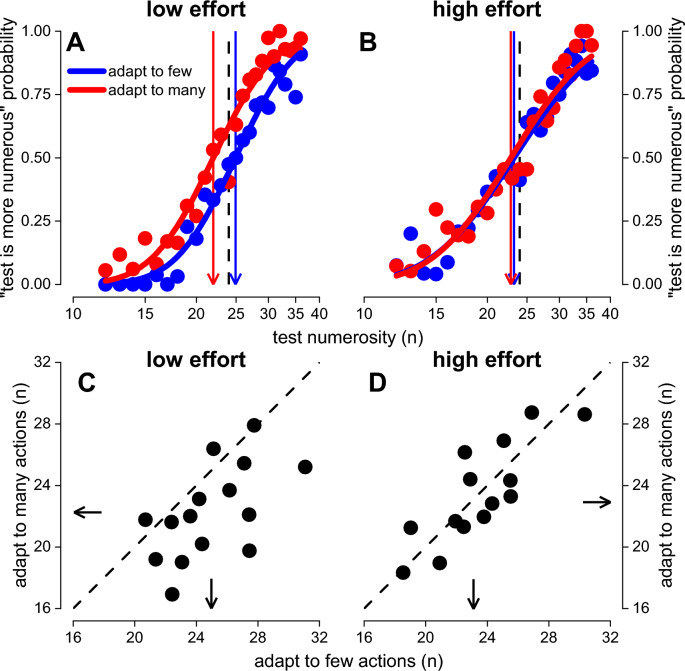
Motor adaptation on visual numerosity perception. **A**, **B** Psychometric curves obtained aggregating the data from all participants obtained in the low (**A**) and high (**B**) effort conditions. Red curves show the results obtained after adapting to many actions, blue curves report the data obtained after adaptation to few actions. **C**, **D** Points of subjective equality (PSEs) estimated by the psychometric curves provided by each individual participant (black dots) in the low (**C**) and high (**D**) effort conditions. Data below the diagonal (equality line) indicates a visual numerosity overestimation after having been adapted to few actions (compared to the many actions adaptation)

We next investigated the effect of adaptation on numerosity perception focusing on the condition-dependent variations in accuracy (i.e., the point of subjective equality, hereafter PSE). Figure 4 reports the psychometric function modelled on the aggregate observer responses (panel A and B) and individual PSEs (C and D), separately for the low (left panels, A and C) and high effort (right panels, B and D) conditions. Panel A shows a replica of the previous results from the literature: under the condition of low motor effort (no additional weight), the number of dots presented in the reference array was underestimated after adapting to many actions (red curve), compared to few actions (blue curve). This result is evident in the gap between the two psychometric curves, with a rightward shift of the blue curve (few action) indicating a relative overestimation of the stimulus presented in the adapted area (i.e. reference stimulus). Panel C shows individual PSEs obtained after the adaptation to many and few actions in the low effort condition. Despite the notable interindividual variability, most of the points lie below the equality line (diagonal dashed line), confirming that, after adapting to few actions, the stimulus presented in the spatial proximity of the adapted location (i.e., reference) was overestimated compared to the condition in which participants adapted to many actions. The results obtained from the high effort condition appear to differ (panel B and D). As shown in panel B, under the condition of high effort, the two psychometric curves were basically superimposed, suggesting similar performance for the two different motor adaptation conditions (many vs few actions). This was confirmed by the distribution of individual PSEs (panel D), where most of the data points lie along the equality line, suggesting no effect of motor adaptation under high motor effort.

**Fig. 5:**
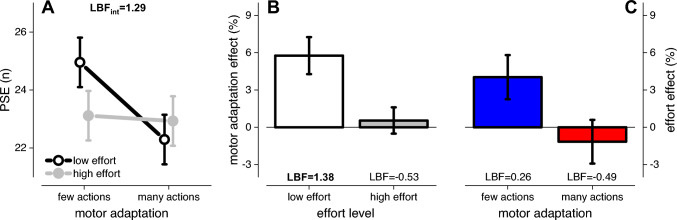
Motor adaptation and effort. **A** Between participants’ average PSEs obtained after adaptation to few or many actions divided by effort conditions (low: black, high: grey). **B** Motor adaptation effect (normalized PSEs shift, see “Materials and methods”) obtained in the low (white) and high (grey) effort conditions. **C** Effect of effort on PSEs after adaptation to few (blue) and many (red) actions. Error bars reports ± 1 s.e.m

To quantitatively estimate these effects, we performed a two-way repeated measure Bayesian ANOVA (2 × 2) on the PSEs, with two effort levels (low and high effort), and two adaptation levels (adapt to few and many actions) as factors. The results revealed that the PSEs are best fitted by the full model, including the two factors and their interaction ($y\sim effort+adaptation+effort:adaptation$; LBF = 1.46), indicating that the bias induced by adaptation to many and few actions differs between effort levels. As shown in Fig. 5A, effort level affected the PSEs greater after the adaptation to few actions (PSEs adapt to few actions: low effort = 24.9, high effort = 23.1), when compared to many actions (low effort = 22.2, high effort = 22.9) as revealed by the analysis of the effects, showing a strong interaction between these two factors (LBF_inclusion_ = 1.29).

To characterize the interaction, we estimated the normalized effect associated with motor adaptation and that associated with physical effort (Fig. 5B, C). For the motor adaptation effect, we computed an index as the difference between PSEs measured in the adaptation to few and many actions, normalized on their sum. Positive values indicate a relative overestimation induced by adaptation to few actions, compared to adapting to many routines (Fig. 5B). On average, in the low effort condition, the evidence for a motor adaptation effect was strong with an average effect of 5.7 ± 1% (Bayesian 2-tailed paired t-test: LBF = 1.38). Conversely, for the high effort condition, the modulation was much weaker (0.5 ± 1%) with substantial evidence for a null effect (Bayesian 2-tailed paired t-test: LBF = − 0.53). The effect related to motor effort was computed as the difference between PSEs measured under low and high effort, normalized by their sum. Positive values indicate a relative overestimation induced by the adaptation to non-strenuous actions (low effort), compared to adapting to effortful actions (high effort). Adapting to few actions resulted in anecdotal evidence for an effect of effort (effort effect: 4.0 ± 1.7%. Bayesian 2-tailed paired t-test: LBF = 0.26, Fig. 5C, blue bar). In other words, adapting to few but strenuous actions just anecdotally reduced the overestimation effect, as compared to few non-strenuous actions. Conversely, when adapting to many actions, the effect of effort was almost zero (− 1.1 ± 1.7%) and with substantial evidence for the null effect (Bayesian 2-tailed paired t-test: LBF = − 0.49, Fig. 5C, red bar).

### Experiments 3: estimation of self-produced actions

The results of the Experiment 2 obtained in the “adaptation to few” actions suggested (anecdotal) evidence for an effect of effort, and a null effect in the “adaptation to many actions”. The effect in the “adaptation to few” actions, while small was sufficient to annul the overall adaptation effect, indicating that effort was integrated (Fig. 4B, C). While the null effect in the “adaptation to many actions” could be reasonably due to a ceiling effect, the effect found in the “adaptation to few actions” does not have an equally plain explanation. One possibility is that effort might have introduced a generalized bias in actions estimation, so that individuals overestimate the number of actions performed under high physical effort conditions. In other words, adapting to few strenuous actions might have reduced the expected visual overestimation because the number of actions were “sensed” as more than they physically were. We tested this possibility in a dedicated experiment. Participants were asked to tap at a relatively slow pace (without counting), mirroring the tapping rate of the adaptation to few actions as in Experiment 2. When the fixation point changed from green to red, participants stopped the sequence of actions and estimated their number. The number of performed actions was computed online by an infrared device. Similarly to Experiment 2, in separate blocks, we applied (high effort condition) or did not apply (low effort condition) a 0.75 kg wristband. If the sensorimotor number system is affected by physical effort, we should observe an overestimation under the high effort condition, relatively to the low effort one.

As a first check, we ensured that the tapping frequency was similar across effort conditions, and comparable to that reported in Experiment 2, when adapting to few actions. On average, the tapping rates for low and high effort condition were 1.4 ± 0.15 and 1.3 ± 0.12 Hz, respectively. Bayesian paired t-test revealed no difference between effort conditions (LBF = − 0.54). Additionally, Bayesian two sample t-test comparing tap rate among the two experiments and confirmed no difference in tapping frequency between adaptation to few actions in Experiment 2 and Experiment 3 (LBF = − 0.45).

**Fig. 6:**
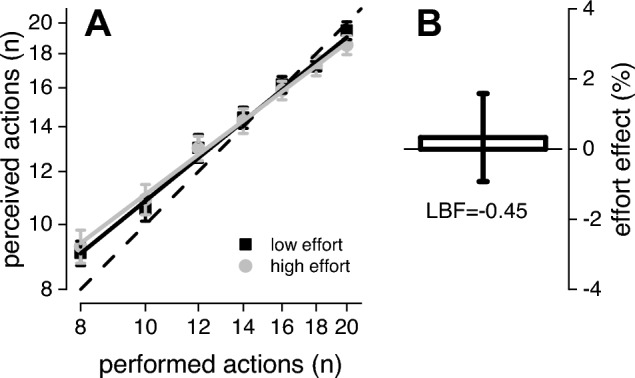
Numerical estimation of self-produced actions. **A** Between participants’ average numerical estimates of self-produced actions obtained wearing (high effort, light grey) or not wearing (low effort, dark grey) a 0.75 kg wristband. Lines report best linear fit. **B** Normalized effect of effort on numerical estimates. Error bars reports ± 1 s.e.m

Figure 6A shows the average estimates of self-produced actions obtained wearing (high effort, light grey) or not wearing (low effort, dark grey) the 0.75 kg wristband. A first visual inspection qualitatively indicates that both conditions led to similar (and fairly accurate) estimates, indicating no effect of effort on the perceived number of self-produced hand actions (at least with this setup). Repeated two-way measure Bayesian ANOVA on numerical estimation, with 2 levels of effort (low/high) and 7 target numbers (8–20, in steps of 2) as factors, indicated that the best model was the one including the number of taps only (LBF = 7.3), indicating that participants were correctly performing the task. The analysis of the effects confirmed no effect of effort (LBF_inclusion_ = − 0.47) and no interaction (LBF_inclusion_ = − 0.73).

Finally, we additionally computed an index of effort effect as the difference between the number of perceived taps in the low vs high effort condition, normalized by their sum. The effect was null for all tested number of taps below 20 (LBF = − 0.56, − 0.39, − 0.59, − 0.56, − 0.51, − 0.58, for 8, 10, 12, 14, 16, 18 taps, respectively), with no definitive evidence for the 20-tap condition (LBF = 0.02). To summarize the data, we averaged these effort effects across number of taps (Fig. 6B), revealing no influence of effort (0.3 ± 1.2%, LBF = − 0.58) on the estimates of self-produced actions, making it unlikely that it accounts for the perceptual distortion on perceived numerosity reported in Experiment 2.

## Discussion

The aim of this study was to investigate whether the sensorimotor number system is selective to the number of actions or also integrates non-numerical motor parameters. More specifically, we investigated the role of physical effort, a parameter positively correlated with the number of actions, in modulating the link between hand-actions and visual numerosity perception. The results of Experiment 1 shows that physical effort, in the absence of actions (passive effort) did not affect visual numerosity estimations, suggesting a preferential link between sequential actions and numerosity. The results of Experiments 2 shows that physical effort is integrated during a specific motor adaptation condition, namely when participants adapt to few actions (but not when adapting to many actions). Finally, Experiments 3 demonstrated that while performing sequential hand actions physical effort is discarded with the system providing accurate estimates of the number of self-produced actions whatever the associated effort.

The evidence for the existence of a sensorimotor number system in humans is largely based on results obtained from adaptation techniques. For instance, after a short period of motor adaptation, during which participants perform few hand-tappings, the perceived numerosity of an array of dots presented near the adapted region is overestimated, while underestimated after many actions (Anobile et al., 2016; Maldonado Moscoso et al., 2020; Togoli et al., 2020; Yang et al., 2024). However, since performing many actions requires more physical effort than few actions, it is difficult to disentangle the relative contribution to numerosity adaptation of the number of actions performed during the adaptation phase from the overall physical effort. To disentangle these possibilities, in the first experiment, we manipulated the physical effort in absence of any sequential action. This experiment aimed at proving whether passive effort was sufficient to modulate visual numerosity representations. In contrast to previous studies investigating the link between action and numerosity, which typically require a motor-adaptation phase consisting of the repetition of sequential actions (Anobile et al., 2016; Maldonado Moscoso et al., 2020; Togoli et al., 2020; Yang et al., 2024), here participants held one hand steady for some time below the screen (in the spatial correspondence of one stimulus) passively holding a weight (or not), and then we presented two arrays of dots and asked participants to discriminate their numerosity. In other words, we measured the influence of physical effort independently from the occurrence of sequential actions (passive effort). The results showed a null effect, with the visual array presented at the steady hand position, equally perceived among the low and high effort conditions. In this experiment, the additional weight was 3 kg. This choice (arbitrary and based on pilot data) was made to simulate and likely exceed the effort experienced by performing sequential actions (the common motor adaptation procedure). The null effect of passive effort is in line with the idea that the execution of sequential actions is necessary to activate the sensorimotor system, suggesting a good level of numerical selectivity.

However, effort might still be integrated in the encoding of visual numerosity when self-produced actions are executed and in turn affect numerosity estimates. To test this possibility, in the second experiment we exploited the classical motor adaptation to sequential actions. Together with the number of actions performed during the adaptation phase (few or many), we also manipulated the effort associated with the hand actions. This was done by having participants wear or not wear a 0.75 kg wristband on the tapping hand. The adaptation to “few actions” was the only condition to provide some (even if anecdotal) evidence for an effect of effort by showing a reduction in the visual overestimation effect when actions were made more strenuous. This effect, despite being small in magnitude was sufficient to cancel out the overall adaptation effect. This specific interaction between effort and motor adaptation could indicate that when the system is overstimulated (adapted) by strenuous actions it considers the number of produced actions to be more numerous than they physically are, resulting in a subsequent reduced visual overestimation induced by adaptation to few actions as this condition would be experienced as more similar to adaptation to “many actions”.

To investigate this possibility, we ran a third experiment in which we asked participants to verbally estimate the number of actions (same tapping as in Experiment 2) they had produced. In this case, the application of a weight to the wrist (as in Experiment 2) showed no effect on the estimation of the number of actions executed, with virtually identical estimates obtained with or without the additional weight. This last result suggests that the small effect of effort found in Experiment 2 was unlikely due to an erroneous estimate of the number of actions produced during the adaptation phase. However, it should be remarked that the two tasks (verbal estimation vs adaptation) are not completely comparable. While in Experiment 2 we did not ask for an explicit estimation of the number of performed actions, in Experiment 3 we did. The null effect found in Experiment 3 could therefore reflect a higher robustness to non-numerical covariates in the case of explicit tasks, compared to implicit (adaptation) processing. This sort of task-dependent susceptibility to covariate parameters has been recently advanced in the domain of time (Petrizzo et al., 2023) and visual numerosity (Lourenco & Aulet, 2023) perception. A further factor that should be considered here is the level of perceived effort. In this regard, we do not have any objective measurements of this parameter. The choice to use a weighted wristband of 0.75 or 3 kg stems from pilot data. While piloting the experiments, we realized that these levels of effort were reasonably acceptable to participants, allowing for the administration of an adequate number of trials (necessary to robust measures) without the necessity to interrupt the trials-blocks. At the same time, these weights induced a robust physical effort as confirmed by all participants of the piloting phase. Indeed, all participants reported experiencing a significant "fatigue" during the task execution with the weight on their hand and required breaks between the blocks of trials. Although these (indirect) indications are somehow reassuring on the effectiveness of the weight used, we cannot rule out the possibility that the use of heavier weights might reveal some effects not measured here.

Taken together, the results suggest that the mechanism combining action and numerosity perception can disentangle motor/sensorimotor numerical information and effort. We observed a null effect of effort on visual numerosity when no actions were involved, indicating that the latter is not sufficient per se to yield any reliable distortions of visual numerosity. A null effect of effort was also observed when participants estimated the number of self-produced actions, indicating that the motor numerical information is also robust to this non-numerical parameter. The only evidence for a possible integration of effort and the number of actions was observed when participants adapted to few actions before estimating visual arrays. The robustness of visual numerosity perception to physical effort is broadly in line with a recent study showing that while running duration but not visual numerosity is distorted (Petrizzo et al., 2022) compared to a resting state. To the best of our knowledge, these are the only two investigations on the role of physical effort on numerosity perception, calling for further works.

## Conclusions

To better understand the mechanisms’ combining actions and numerosity perception, in the current study we assessed the role of a non-numerical parameter associated with self-produced motor routines: physical effort. The results indicate that passive effort is discarded suggesting that actions are necessary to activate the system. Similarly, effort is also discarded when estimating the number of self-produced hand actions. However, the results show that during motor adaptation, and more specifically when adapting to “few” actions, physical effort modulated the adaptation effect suggesting that under certain circumstances it could be integrated and used by the system. Overall, the current results are generally in line with previous evidence showing that response duration, another non-numerical motor parameter, is discarded by the sensorimotor number system that indeed seems to combine genuinely the number of sequentially performed actions with that of perceived stimuli (Anobile et al., 2024; Kirschhock & Nieder, 2022). Understanding the functional architecture of this newly discovered system, whether it is selective to the number of actions, represents fundamental pieces of information necessary to pave the way for further investigations, for example on its functional role. The current results contribute to this general goal clearly calling for further investigations.

## Data Availability

The datasets generated and analysed during the current study are available in the Zenodo repository, https://zenodo.org/records/10911429.
